# Review of machine learning for optical imaging of burn wound severity assessment

**DOI:** 10.1117/1.JBO.29.2.020901

**Published:** 2024-02-15

**Authors:** Robert H. Wilson, Rebecca Rowland, Gordon T. Kennedy, Chris Campbell, Victor C. Joe, Theresa L. Chin, David M. Burmeister, Robert J. Christy, Anthony J. Durkin

**Affiliations:** a University of California, Irvine, Beckman Laser Institute and Medical Clinic, Irvine, California, United States; b University of California, Irvine, Department of Medicine, Orange, California, United States; c University of California, Irvine, Health Policy Research Institute, Irvine, California, United States; d UC Irvine Health Regional Burn Center, Orange, California, United States; e UC Irvine Medical Center, Orange, California, United States; f Uniformed Services University of the Health Sciences, School of Medicine, Bethesda, Maryland, United States; g UT Health San Antonio, Military Health Institute, San Antonio, Texas, United States; h University of California, Irvine, Department of Biomedical Engineering, Irvine, California, United States

**Keywords:** machine learning, burn severity, burn assessment, burn wound, debridement, optical imaging, artificial intelligence, tissue classification

## Abstract

**Significance:**

Over the past decade, machine learning (ML) algorithms have rapidly become much more widespread for numerous biomedical applications, including the diagnosis and categorization of disease and injury.

**Aim:**

Here, we seek to characterize the recent growth of ML techniques that use imaging data to classify burn wound severity and report on the accuracies of different approaches.

**Approach:**

To this end, we present a comprehensive literature review of preclinical and clinical studies using ML techniques to classify the severity of burn wounds.

**Results:**

The majority of these reports used digital color photographs as input data to the classification algorithms, but recently there has been an increasing prevalence of the use of ML approaches using input data from more advanced optical imaging modalities (e.g., multispectral and hyperspectral imaging, optical coherence tomography), in addition to multimodal techniques. The classification accuracy of the different methods is reported; it typically ranges from ∼70% to 90% relative to the current gold standard of clinical judgment.

**Conclusions:**

The field would benefit from systematic analysis of the effects of different input data modalities, training/testing sets, and ML classifiers on the reported accuracy. Despite this current limitation, ML-based algorithms show significant promise for assisting in objectively classifying burn wound severity.

## 1 Introduction

Incorporating emerging technologies into the clinical workflow for the early staging of burn severity may provide a crucial inroad toward improved diagnostic accuracy and personalized treatment.^1^ Early knowledge of the severity of the burn gives the clinician the ability to discuss treatment options and prognostication for hospital stays, healing, and scarring. Within the spectrum of burn severity, superficial partial thickness burns often do not require skin grafting but can be managed with daily wound care or covered with various synthetic or biologic dressings. Full thickness burns typically require skin grafting because they will take >3 weeks to heal and can result in symptomatic and constricting scars. Deep partial thickness burns can act like full thickness burns and require skin grafting. Distinguishing the burn severity along the spectrum can be difficult early after injury and is subjective when based on previous clinical experience. Modalities that allow for additional objective data promptly after injury helps the clinician to manage the wound properly to enable healing of the damaged tissue and reduce infection, contracture, and other unfavorable outcomes.^2^ In many cases, some portions of a burn wound need grafting but other portions do not, so it is vital to develop imaging techniques that can spatially-segment tissue regions with deeper burns from locations where the burn is more superficial. For the above reasons, burn wound assessment is a prime example of an application for which the combination of optical imaging devices and machine learning (ML) algorithms has recently made notable progress toward translation to clinical care.

**Fig. 1:**
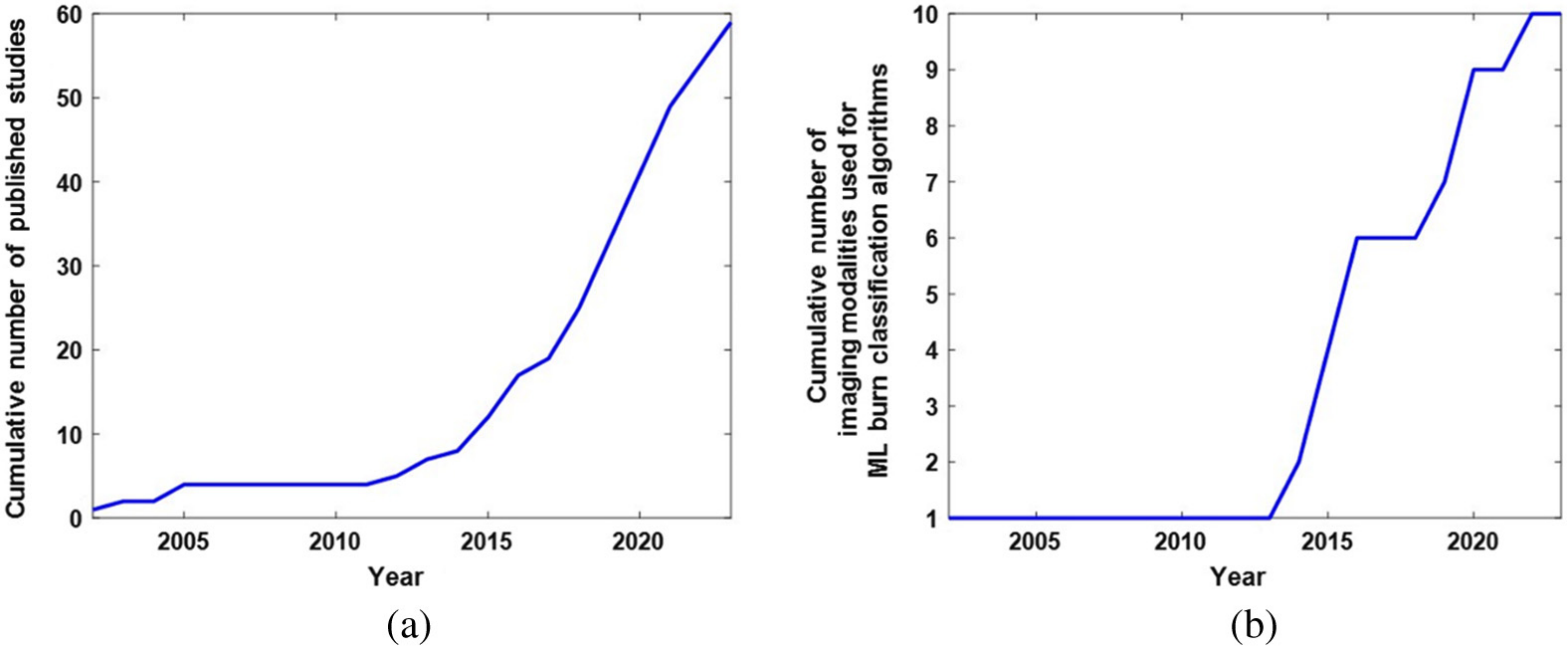
Over the past decade, there has been a rapid increase in the number of studies developing machine learning approaches for burn wound classification using imaging data. (a) Cumulative number of published studies on ML burn classification methods using imaging data as a function of time over the past two decades. A progressively steeper increase in the cumulative number of publications is observed, especially over the past decade. (b) Cumulative number of different imaging modalities employed to train ML-based burn wound classification algorithms in published studies, plotted as a function of time over the past two decades. As with the cumulative number of published studies, the cumulative number of imaging modalities used in these applications has increased sharply over the past decade.

ML algorithms are becoming ubiquitous in a wide variety of disciplines. In the medical field, ML is attractive due to its potential for objective classification of disease and injury, categorization of stage of disease and severity of injury, informing treatment, and prognosticating clinical outcomes. ML can conveniently manage and interpret high-dimensional multimodal clinical datasets to facilitate the translation of these data into powerful tools to help inform clinical decision making.^3^^–^^5^ Over the past decade, numerous research groups have begun to test the efficacy of merging ML algorithms with imaging technologies for classifying burn wounds.^6^^–^^12^ The input data in these studies is frequently obtained from standard red, green, and blue (RGB) color images. However, other emerging techniques (e.g., multispectral imaging, optical coherence tomography, spatial frequency domain imaging, and terahertz imaging) are being developed for this application as well. This literature is growing at a rapid rate, both in terms of the number of new reported studies and the range of different technologies used for obtaining input data to train ML classifiers (Fig. 1). The “ground-truth” categorization of burn severity used for training the algorithms is typically the clinical impression, which is regarded as the diagnostic/prognostic “gold standard” but can be incorrect in ∼20% to 50% of cases.^13^^–^^16^ The outputs of the algorithm are typically (1) the segmentation of burned versus unburned tissue and (2) the classification of depth or severity of the burn. The literature encompasses studies both in preclinical animal models and in clinical settings.

The purpose of this review article is to provide an overview of the progress thus far in the combination of ML algorithms with different optical imaging modalities to assist with burn wound assessment. The review is organized according to the type of imaging modality used as input data for different ML studies, which are summarized in Table 1. Section 2 focuses on ML techniques using data from conventional color photography. Section 3 discusses the use of ML algorithms for analyzing multispectral (and hyperspectral) imaging data. Section 4 analyzes the use of other modalities of imaging data (optical coherence tomography, ultrasound, thermal imaging, laser speckle and laser Doppler imaging, and terahertz imaging) with ML technology. Section 5 provides a summary of the findings of this review and briefly discusses potential future directions.

**Table 1:** Summary of different ML-based burn classification studies using imaging data as inputs to the ML algorithms. Modality, data processing techniques, ML classifiers, validation procedures, and reported accuracy values are shown in the columns of the table.

Data modality	Studies	Pre-processing	ML classifier	Validation methods	Accuracy
Digital color	17–52	E.g., L, a, b; texture analysis	E.g., SVM, LDA, KNN, deep CNN	E.g., k-fold CV; separate test set	80.9% +/− 6.4% without deep learning; 86.2% +/− 9.8% with deep learning (see Fig. 7)
Multispectral	53	Outlier detection	SVM, KNN	tenfold CV	76%
	54		LDA, QDA, KNN		68%–71%
	55		CNN		Sensitivity = 81%; PPV = 97%
Hyperspectral	56	Denoising	Unsupervised segmentation	Comparison between segmentation and histology	Not reported
Multispectral SFDI	57	Calibrated reflectance	SVM	tenfold CV	92.5%
Digital color + multispectral	58,59	Texture analysis, mode filtering	QDA	twelvefold CV (in Ref. 59)	78%
	60		QDA + k-means clustering	34-fold CV	24% better than QDA alone for identifying non-viable tissue
	61	Outlier removal using Mahalanobis distance			
OCT	62	OCT and pulse speckle imaging	Naïve Bayes classifier		ROC AUC = 0.86
	63	A-line, B-scan, and phase data	Multilevel ensemble classifier	tenfold CV	92.5%
	64	Eight OCT parameters	Linear model classifier	Test set	91%
Ultrasound	65 (*ex vivo*)	Texture analysis	SVM and kernel Fisher		93%
	66 (*in situ* postmortem)	B-mode ultra sound data	Deep CNN		99%
Thermography	67	Thermography and multispectral	CNN pattern recognition	Training, validation, and test sets	Precision = 83%
	68	Temperature difference relative to healthy skin	Random forest	Training and validation sets	85%
Blood Flow	69	LSI	CNN		>93%
Terahertz Imaging	70,71	Wavelet denoising, Wiener deconvolution	SVM, LDA, Naïve Bayes, neural network	Fivefold CV	ROC AUC = 0.86-0.93
	72	Permittivity	Three-layer fully connected neural network	Fivefold CV	ROC AUC = 0.93

Note: PPG, photoplethysmography; OCT, optical coherence tomography; LSI, laser speckle imaging; SVM, support vector machine; LDA, linear discriminant analysis; KNN, K-nearest neighbors; QDA, quadratic discriminant analysis; CNN, convolutional neural network; CV, cross-validation; ROC, receiver operating characteristic; AUC, area under curve.

## 2 Use of ML with Color Photography

To date, the majority of studies that have examined the use of ML to categorize burns have done so using color photography data as inputs. These studies date back nearly two decades^17^^–^^20^ but have become significantly more numerous over the past decade, especially during the past 5 years.

### 2.1 Early Studies

The first reported work in this area^17^^–^^20^ analyzed images in the (L*,u*,v*) color space, which is representative of the human perception of color. Parameters related to texture and color were extracted from the images and used as inputs to the ML algorithm, which was based on a neural network known as a Fuzzy-ARTMAP (fuzzy logic merged with adaptive resonance theory for analog multidimensional mapping). This approach was tested on clinically obtained color images of full thickness, deep dermal, and superficial dermal burns. In Refs. 18 and 19, with a dataset of 62 images, the ML classification method provided a mean overall accuracy (across all three categories) of 82%, relative to the “gold standard” of visual inspection by burn care experts. In Ref. 20, with a dataset of 35 images, the mean overall accuracy of the ML classifier was 89%.

Additional research began to emerge roughly half a decade later. A 2012 study^21^ compared support vector machine (SVM), K-nearest neighbor (KNN), and Bayesian classifiers, using image segmentation to identify burn regions and input parameters from texture analysis and h-transformed data to classify burn severity. Fourfold cross-validation provided the highest classification accuracy of burn severity (89%) when SVM was used. A “blind test” of the SVM provided a classification accuracy of 75% for distinguishing between grades of burn severity. A 2013 report^22^ compared SVM, KNN, and template matching (TM) algorithms for classifying three different burn severity categories (superficial, partial thickness, and full thickness) in patients with a range of different demographic characteristics (age, gender, and ethnicity). Using a sample size of 120 images (40 superficial burns, 40 partial thickness burns, and 40 full thickness burns), the overall classification accuracy was 88% for the SVM, 75% for the TM, and 66% for the KNN.

Another 2013 study^23^ used multidimensional scaling (MDS) to quantify features of color images that were related to burn depth. Parameters from this model were input into a KNN algorithm for classification. The KNN provided 66% accuracy for distinguishing between three different burn depth categories (superficial dermal, deep dermal, and full thickness) and 84% accuracy for distinguishing between burns requiring grafts versus burns not needing grafting. When principal component analysis (PCA) was performed and the three most significant principal components were used as inputs into the KNN, the accuracy decreased to 51% for the three-group classification (superficial dermal, deep dermal, and full thickness) and 72% for the two-group classification (graft needed versus no graft needed). When the MDS parameters were input into an SVM, the accuracy values were 76% and 82% for the aforementioned three-group classification and two-group classification, respectively. A similar study in 2015^24^ reported an accuracy of 80% for distinguishing burns in need of grafts from burns not in need of grafts, when MDS parameters were used in conjunction with an SVM classification algorithm on an independent test dataset of 74 images.

Another 2015 study^25^ used an SVM to classify burns by severity (second degree, third degree, and fourth degree) with 73.7% accuracy when twofold cross-validation was performed. A 2017 report^26^ compared 20 different algorithms for classifying burn severity into three categories (superficial partial thickness, deep partial thickness, and full thickness), using both tenfold cross-validation and an independent test dataset. The highest classification accuracy (73%) obtained using cross-validation was achieved with a simple logistic regression algorithm. Five of the algorithms were identified as the most accurate for classifying burns in the test dataset, and their mean accuracies were all 69%. The low classification accuracy was primarily attributed to difficulties in using the algorithms to distinguish between superficial partial thickness and deep partial thickness burns. This issue was linked to the observation that the superficial partial thickness burns often included some deep partial thickness burn regions, and vice versa, making classification difficult.

### 2.2 Studies from 2019 to 2023: Emergence of Deep Learning Approaches

At the time of this report, the majority of studies on ML methods for classifying color images of burns were published from 2019 to 2023. Although some preliminary burn classification work using digital color images and deep learning technology had been reported prior to 2019,^27^ the period from 2019 to 2023 saw a substantial increase in the use of deep learning approaches for burn wound classification.^28^^–^^45^

**Fig. 2:**
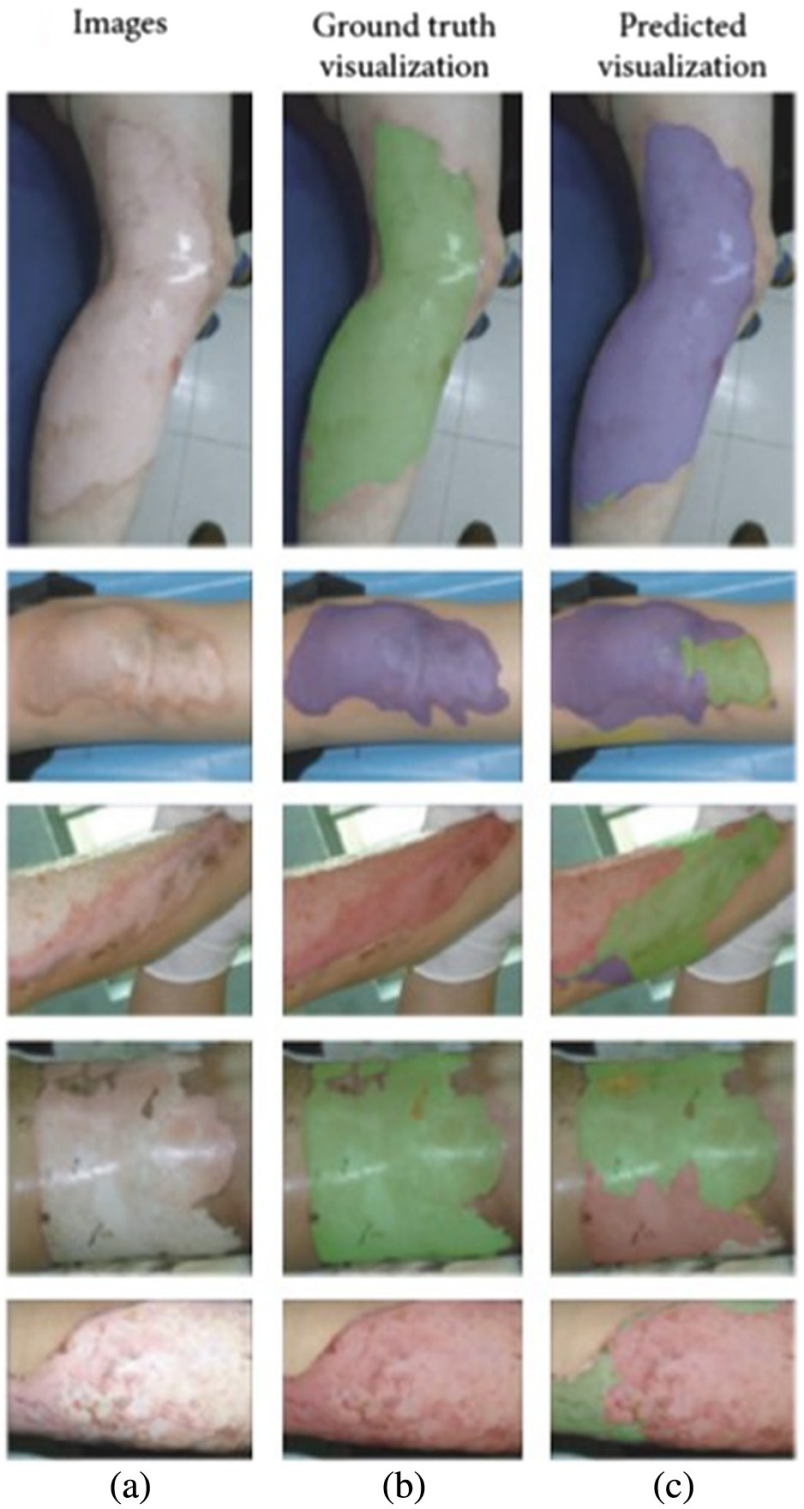
Results of a deep learning algorithm for classifying burn severity using color photography data, mapped across the tissue surfaces of patients. Data from color images (a) were input into a multi-layer deep learning procedure including segmentation and feature fusion. The algorithm classified burn regions (c) as superficial partial-thickness (blue), deep partial-thickness (green), and full-thickness (red). Ground-truth categorization (b) is shown for comparison (adapted from Ref. 34, with permission).

Several studies in this time period used deep learning algorithms to segment images into burned and un-burned regions.^31^^,^^34^^,^^35^^,^^38^^–^^40^ A 2019 study^31^ used 1,000 images to train a mask region with a convolutional neural network (Mask R-CNN) algorithm, comparing several different underlying network types and obtaining a maximum accuracy of 85% for identifying burn regions in images of different severities of burns. Another 2019 study^38^ used deep learning with semantic segmentation to distinguish between burn, skin, and background portions of images. Two 2021 studies^34^^,^^35^ (Fig. 2) used deep learning algorithms to segment burned versus un-burned tissue to determine the total body surface area (TBSA) that was burned.

A 2019 study^28^ used deep CNN based approaches for burn classification, comparing four different networks. The ResNet-101 deep CNN algorithm distinguished between four different burn categories (superficial partial-thickness, superficial-to-intermediate partial-thickness, intermediate-to-deep partial-thickness, and deep partial thickness to full thickness) with a mean accuracy of 82% when tenfold cross-validation was performed. In another study by this same group,^29^ a tensor decomposition technique was employed to extract parameters related to texture for input into a cluster analysis algorithm that segmented the images into three categories (non-tissue “background,” healthy skin, and burned skin) with a sensitivity of 96%, positive predictive value of 95%, and faster computation time than other image analysis techniques (e.g., PCA). A third report from this group^30^ used digital color images acquired with a commercial camera specialized for tissue imaging (TiVi700, WheelsBridge AB, Sweden) that uses polarization filters. The polarized images were used to train a U-Net deep CNN for distinguishing between four different burn severities (superficial partial-thickness, superficial-to-intermediate partial-thickness, intermediate-to-deep partial-thickness, and deep-partial thickness to full-thickness, which is defined based on healing times). The accuracy of this technique was 92% when a separate test set (consisting of data not included in the training set) was used.

A 2020 study^32^ used a ResNet-50 deep CNN to classify burns into three levels of severity (shallow, moderate, and deep, which is based on the time/intervention required to heal) with an overall accuracy of ∼80% when applied to a separate test dataset. Another 2020 study^41^ used a deep CNN to classify burns as superficial, deep dermal, or full thickness in a separate test dataset with an average accuracy of 79%. A third study from 2020^33^ employed separate SVMs, trained with features identified by deep CNNs, for each body part examined (inner forearm, hand, back, and face), achieving burn severity (low, moderate, and severe) classification accuracy of 92% and 85% for two different test datasets. A 2021 report^42^ used deep neural network and recurrent neural network approaches to classify burns as first, second, or third degree with accuracies of 80% and 81%, respectively.

A 2023 study^36^ performed deep learning on color images of patients with a wide range of Fitzpatrick skin types to identify burned regions and classify whether those regions required surgical intervention. Patients were split into two subsets: those with Fitzpatrick Skin Types I-II and those with Fitzpatrick Skin Types III-VI. The classification algorithm employed a deep CNN made available via commercially available software (Aiforia Create, Helsinki, Finland). For distinguishing burns for which surgical intervention was needed from burns that did not require surgical intervention, using a separate test dataset, the area under the receiver operating characteristic curve (ROC AUC) was nearly identical for the dataset from patients with lighter skin (AUC = 0.863) and the dataset from patients with darker skin (AUC=0.875). This result is expected due to the fact that the burn removed the epidermis (where melanin is located). Despite the high AUC, the overall accuracy of the algorithm was 64.5%. Two studies by the same group^43^^,^^44^ used new deep CNN algorithms to classify burn severity (superficial, deep dermal, and full thickness) with >97% accuracy and distinguish between burns in need of grafting and burns not requiring grafts with >99% accuracy, using fivefold cross-validation.

Another 2022 study^37^ compared “traditional” (non-deep-learning-based) ML algorithms with deep learning approaches for classifying images of burns in patients. For distinguishing between first degree (superficial), second degree (partial thickness), and three degree (full thickness) burns in a separate test set, the most accurate “traditional” ML approach was a random forest classifier with an augmented training dataset (accuracy = 80%). For performing the same classification, the most accurate deep learning approach was a deep CNN that used transfer learning from a pre-trained model (VGG16). The accuracy of this algorithm was 96%, considerably higher than the best “traditional” method. A 2021 study^45^ performed a similar comparison, in which a deep learning approach incorporating CNN and transfer learning classified burn images into three categories (superficial dermal, partial thickness, and full thickness) with an accuracy of 87% compared with 82% when an SVM was used.

**Fig. 3:**
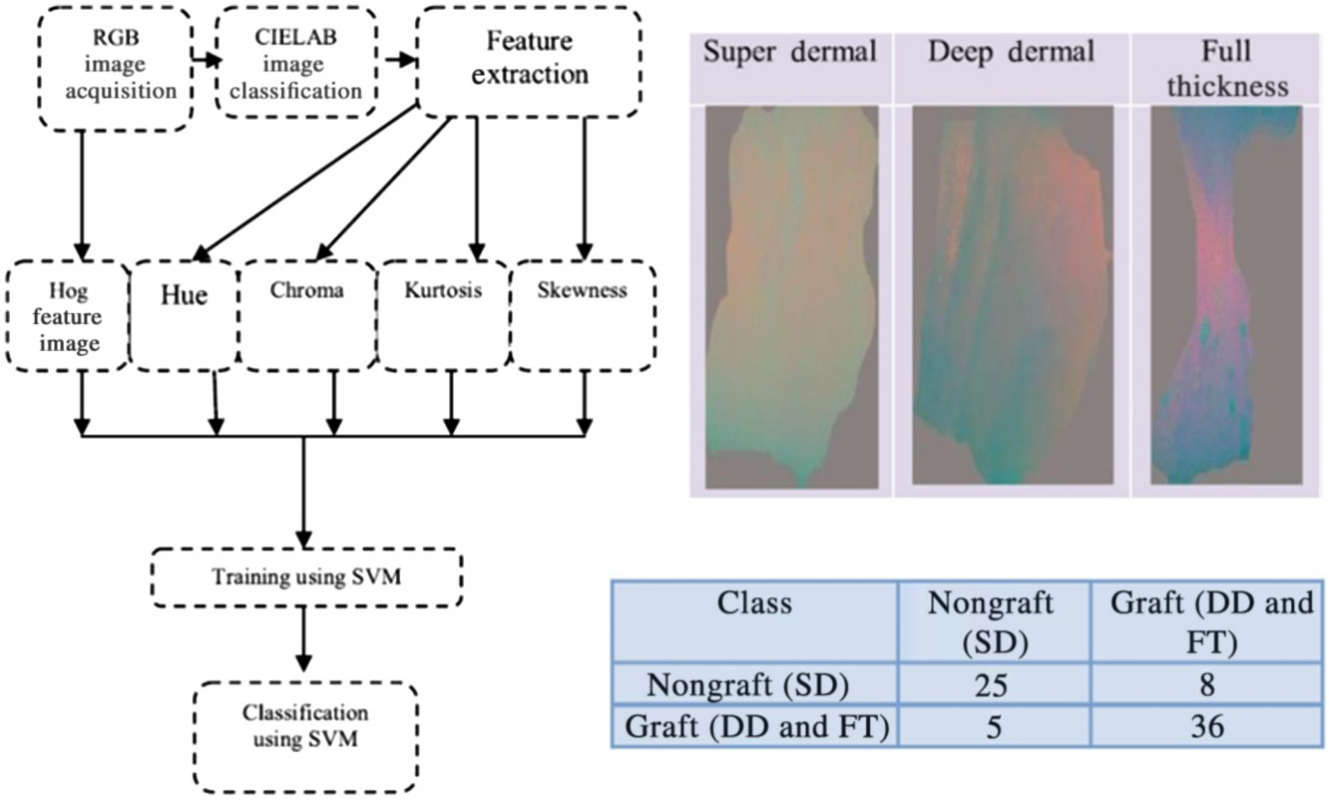
Workflow of human burn severity classification method using parameters obtained from color images. Four different parameters (hue, chroma, kurtosis, and skewness) are extracted from the color images in the CIELAB space. Additionally, the histogram of oriented gradients (Hog) feature is calculated to provide local information about the shape of the region of the tissue that was burned. These parameters are employed to train an SVM to classify the severity of the burn. The combination of L*, a*, and b* parameters shows different types of contrast for different categories of burns (super dermal, deep dermal, and full thickness). For distinguishing super dermal burns (which do not require grafting) from the other two categories (which require grafting), 61 out of 74 burns (82%) were classified correctly (adapted from Ref. 46, with permission).

Over the period from 2019 to 2023, there were also several studies that used “traditional” (non-deep-learning-based) ML algorithms for burn wound classification.^46^^–^^52^ One such study^46^ (Fig. 3) used an SVM trained on a subset of the test set to distinguish between burns requiring a graft versus burns not requiring a graft, with an accuracy of 82%. In a subsequent report,^47^ this same group used a kurtosis metric, obtained following the segmentation of color images via simple linear iterative clustering (SLIC), as an input into an SVM for classifying burns as requiring grafting versus not requiring grafting. Using an open-access database (BURNS BIP-US) to form the training set and test set, the classification accuracy of the SVM was 89%. Another recent study^48^ used a procedure to extract feature vectors (incorporating data related to texture and color) from different regions of color images to categorize the burns as first degree, second degree, or third degree in each region with sensitivity and precision >89% for each category when fourfold cross-validation was used. A recent set of studies^49^^–^^52^ developed burn classification ML algorithms for a diverse range of datasets spanning patients with notably different skin tones. The percentage of studies in the 2019 to 2023 period that used non-deep-learning ML algorithms was significantly less than the pre-2019 period due to the substantially increased prevalence of deep learning techniques.

## 3 Use of ML with Multispectral and Hyperspectral Imaging

### 3.1 ML and Multispectral Imaging

**Fig. 4:**
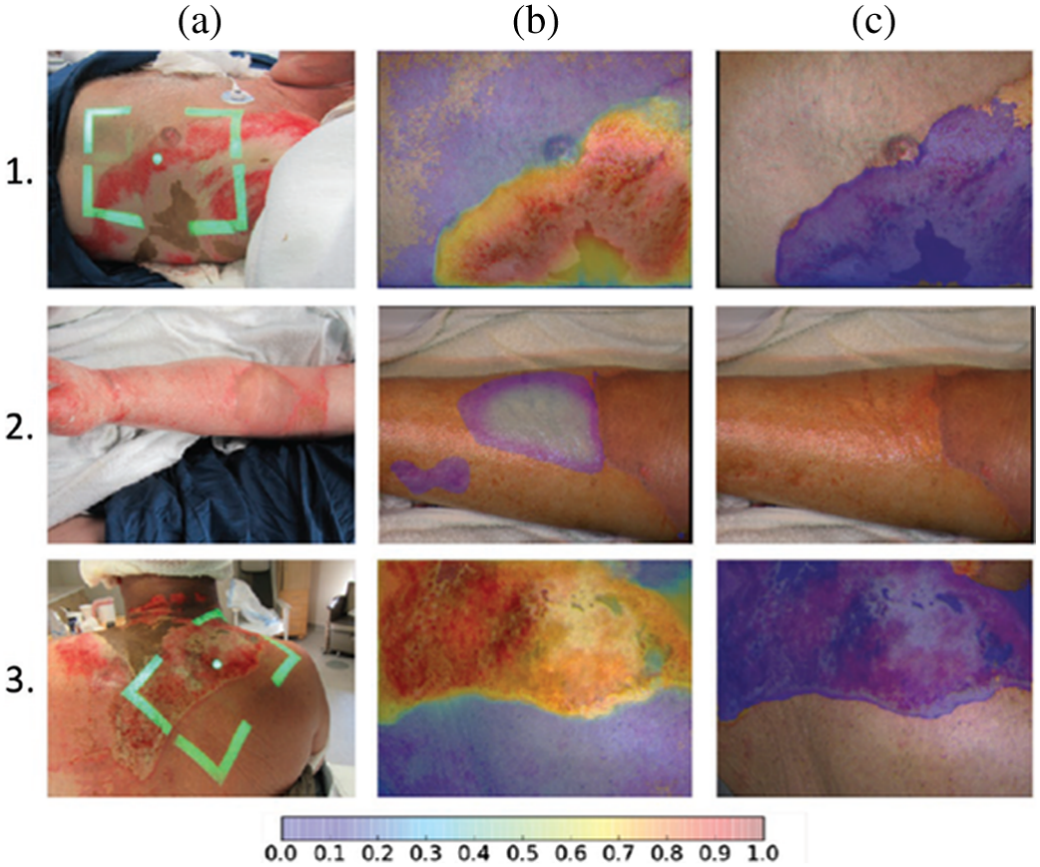
Tissue classification results using multispectral imaging data to train CNN. (a) Digital color images from three burn patients. The patients in Rows 1 and 3 had severe burns; the patient in Row 2 had a superficial burn. (b) A probability map of burn severity, where purple/blue colors represent a low probability of a severe burn and orange/red colors denote a high probability of a severe burn. The clear-appearing region in the middle of burn (2b) represents a set of pixels with probability < 0.05 of severe burn. (c) A segmented probability map in which purple pixels denote a probability of severe burn that exceeds a user-defined threshold. The algorithm performed well at correctly identifying the two severe burns and distinguishing them from the superficial burn (reproduced from Ref. 55, with permission).

Studies over the past decade have merged ML approaches with multispectral imaging to enhance the input datasets used for training the burn classification algorithms. A 2015 report^53^ used a broadband light source and monochrome camera with a filter wheel in front, to acquire images in eight wavelength bands with center wavelengths ranging from 420 to 972 nm. The system was used to image burns in male Hanford pigs, and a maximum likelihood estimation-based algorithm for outlier detection was employed for post-processing. Subsequently, the remaining dataset (with outliers removed) was input into the KNN and SVM algorithms for distinguishing between six different types of tissue (healthy, wound bed, partial injury, full injury, blood, and hyperemia). When a tenfold cross-validation procedure was used, the overall classification accuracy was 76%. The authors noted a particular challenge with classifying blood due to the multiple peaks of its absorption spectrum in the wavelength range imaged. Another recent report^54^ compared the classification accuracy of eight different ML algorithms for differentiating among the aforementioned six tissue categories using multispectral imaging data from male Hanford pigs as inputs. Four of the algorithms (linear discriminant analysis, weighted-linear discriminant analysis, quadratic discriminant analysis, and KNN) had average accuracy values between 68% and 71%, and the other four algorithms (decision tree, ensemble decision tree, ensemble KNN, and ensemble linear discriminant analysis) had average accuracy values ranging from 37% to 62%. A more recent clinical study^55^ (Fig. 4) used a multispectral imager consisting of a light-emitting diode and a camera with a filter wheel including filters centered at eight different VIS-NIR wavelength bands (420, 581, 601, 620, 669, 725, 860, and 855 nm). Patients with three different burn categories (superficial partial-thickness, deep partial-thickness, and full-thickness, as confirmed via biopsy and histopathology) were imaged within the first 10 days following injury. The imaging data were used to train three different CNNs for distinguishing non-healing burns (deep partial-thickness and full-thickness) from all other tissue types. The most accurate CNN (a Voting Ensemble algorithm) provided a sensitivity of 80.5% and a PPV of 96.7% for the aforementioned healing versus non-healing classifications. CNN-based classification was also applied to the subset of burns that had initially been classified as “indeterminate depth” by clinicians at the time they were imaged. For this subset, the sensitivity of the ML algorithm was 70.3% and the PPV was 97.1% for correctly classifying the burns into healing versus non-healing categories.

### 3.2 ML and Hyperspectral Imaging

**Fig. 5:**
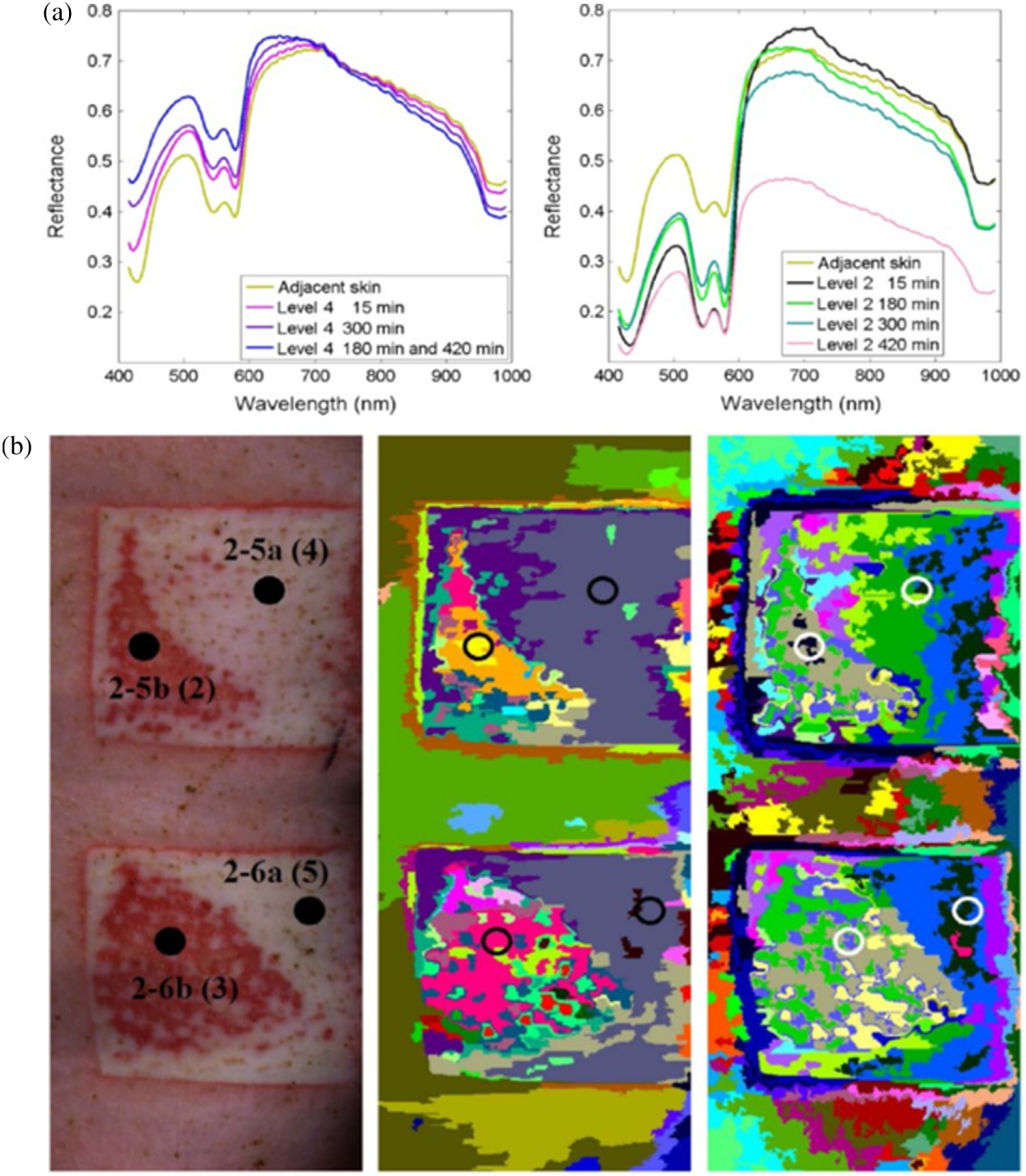
Classification of burn severity using hyperspectral imaging data. (a) Notable differences are seen in the 400 to 600 nm range of the reflectance spectra of more severe burns (Level 4) and less severe burns (Level 2) in a porcine model. These differences are likely attributable to changes in the concentration of hemoglobin (which strongly absorbs light in this wavelength regime) due to different levels of damage to the tissue vasculature. (b) Different burn severities (left column) are classified using two different segmentation algorithms: a spectral-spatial algorithm (center column) and a K-means algorithm (right column) (adapted from Ref. 56, with permission).

ML techniques have also recently been used with hyperspectral imaging systems to incorporate even more robust input datasets into burn classification algorithms. A 2016 study^56^ (Fig. 5) used two different cameras (one in the 400 to 1000 nm spectral range and another in the 960 to 2500 nm spectral range) to image burns in Noroc pigs (50%/25%/25% hybrid of Norwegian Landrace, Yorkshire, and Duroc). Both cameras performed line scans using push-broom techniques. The resulting hyperspectral imaging data were input into an unsupervised algorithm for performing image segmentation in both the spatial and spectral dimensions. This segmentation technique was found to compare favorably with K-means segmentation for distinguishing different burn severities. A recent case study^73^ performed hyperspectral (400 to 1000 nm) imaging of a human partial thickness burn and used principal component analysis and a spectral unmixing technique to categorize different types of tissue.

### 3.3 Combining Multispectral Imaging with Color Image Analysis and Photoplethysmography

A set of four recent studies^58^^–^^61^ used a combination of (1) multispectral imaging (eight wavelengths, ranging from 420 to 855 nm), (2) texture analysis of color image data, and (3) photoplethysmography (PPG) for classifying burn wounds. The initial work of this group^58^^,^^59^ demonstrated that inputting data from these three modalities into a quadratic discriminant analysis (QDA) ML algorithm provided an accuracy of 78% for classifying four different tissue types (deep burn, shallow burn, viable wound bed, and healthy skin) in Hanford pigs. This represented a dramatic improvement over the classification accuracy obtained using just PPG (45%), a notable improvement over that obtained with only color image texture analysis parameters (62%), and a slight improvement over the result from using only multispectral imaging data (75%). These values of overall accuracy were impacted significantly by the fact that the classification algorithms typically yielded accuracy values below 50% for classifying shallow burns. In a follow-up study by the same group,^60^ the QDA technique (which is supervised) was combined with a k-means clustering algorithm (which is unsupervised) to classify human burn wounds. The combination of k-means clustering and QDA resulted in an overall mean accuracy of 74% for distinguishing between viable and non-viable skin compared with 70% when only QDA was used. An additional report^61^ incorporated a post-processing procedure using Mahalanobis distance calculations to help remove outliers, but it only used multispectral and color images (not PPG). This algorithm provided an accuracy of 66% for classifying non-viable human tissue compared with 58% when classification was performed without the outlier-removal routine. Thus, in this study, the omission of PPG data likely contributed to the decreased classification accuracy.

### 3.4 Multispectral Spatial Frequency Domain Imaging

**Fig. 6:**
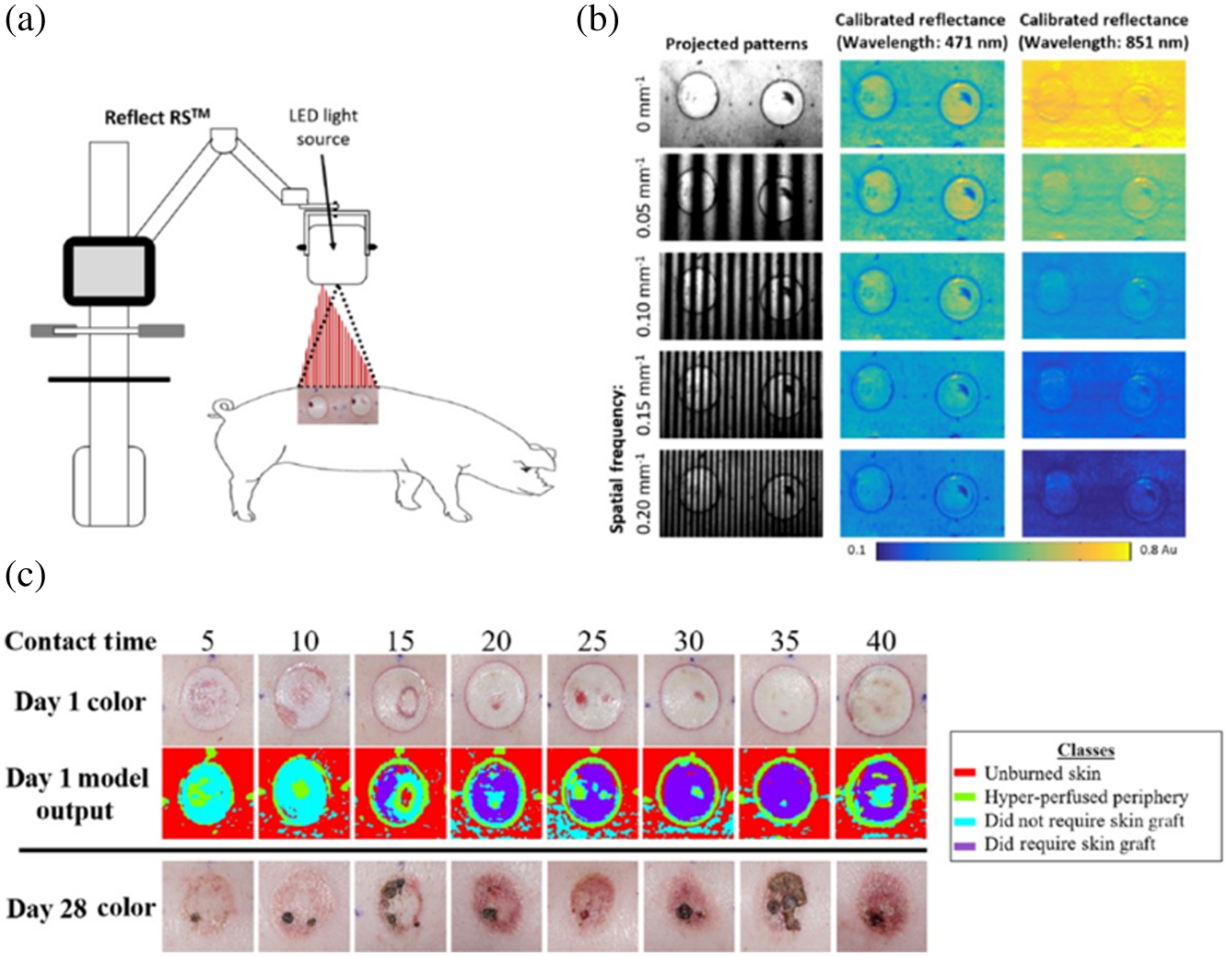
ML-based classification of burn severity in a preclinical model using multispectral spatial frequency domain imaging (SFDI) data. (a) A commercial device (Modulim Reflect RS™) projected patterns of light with different wavelengths and spatially modulated (sinusoidal) patterns onto a porcine burn model and detected the backscattered light using a camera. (b) The backscattered images at the different spatial frequencies were demodulated and calibrated to obtain reflectance maps at each wavelength. The relationship between reflectance and spatial frequency was different at the different wavelengths (e.g., 471 nm versus 851 nm, as shown here). (c) The reflectance data at each wavelength were used to train an SVM to distinguish between four different types of tissue (unburned skin, hyper-perfused periphery, burns that did not require grafting, and burns that required grafting). The ML algorithm reliably distinguished more severe burns (originating from longer thermal contact times) from less severe burns. When using a tenfold cross-validation procedure, the overall diagnostic accuracy of the method was 92.5% (adapted from Ref. 57, with permission).

A recent report by our group^57^ (Fig. 6) used an emerging technique called multispectral spatial frequency domain imaging for ML-based burn wound classification in a Yorkshire pig model. In this study, light with combinations of eight different visible to near-infrared wavelengths (470 to 851 nm) and spatially modulated sinusoidal patterns of five different spatial frequencies (0 to $0.2\;{\mathrm{mm}}^{-1}$) was used to image different severities of pig burns. The rationale behind using the spatially modulated light was that the different spatial frequencies are known to have different mean penetration depths into the tissue, thereby potentially providing more detailed information about the extent of burn-related tissue damage beneath the surface. Calibrated diffuse reflectance images from different combinations of the wavelengths and spatial frequencies were then input into an SVM to classify the severity of the burns. When images from all 40 combinations of the five spatial frequencies and eight wavelengths, acquired 1 day post-burn, were used to train the SVM, burn severity classification (no graft required versus graft required) with an accuracy of 92.5% was obtained for a tenfold cross-validation. For comparison, when only the unstructured (spatial frequency = 0) images at the eight different wavelengths were used as inputs (to mimic standard multispectral imaging), the accuracy of the classification algorithm was 88.8% for the same cross-validation procedure.

## 4 Use of ML with Other Imaging Techniques

### 4.1 Optical Coherence Tomography (OCT)

Several studies have used OCT, either alone or in combination with another technology, to measure data for input into ML burn classification algorithms. A 2014 report^62^ acquired pulse speckle imaging (PSI) data along with OCT 1 h post-burn to distinguish full-thickness, partial-thickness, and superficial burns in a Yorkshire pig model. Using a Naïve Bayes classifier with data from the combination of these two techniques yielded an area under the receiver operating characteristic curve (ROC AUC) of 0.86 for accuracy of classifying the three categories of burns, compared with 0.62 when only OCT data were used and 0.78 when only PSI data were used. A 2019 study^74^ combined OCT with Raman spectroscopy (using laser excitation at 785 nm) to inform ML-based classification of full-thickness, partial-thickness, and superficial partial-thickness porcine burns *ex vivo*. Parameters from Raman spectroscopy measured data related to tissue biochemical composition, specifically, the NCαC/CC proline ring ratio ($943/971\;{\mathrm{cm}}^{-1}$), CH bending/Amide III ratio ($1300/1268\;{\mathrm{cm}}^{-1}$), and ${\mathrm{CH}}_{2}$ bending/Amide ICO stretch ratio ($1450/1660\;{\mathrm{cm}}^{-1}$). Parameters from OCT provided data about the tissue structure. The combination of OCT and Raman spectroscopy data resulted in an ROC AUC of 0.94 for classifying the three different types of burns. A recent study on human skin *in vivo*^63^ used parameters measured with polarization sensitive OCT (phase information, in addition to A and B scans) for a multi-level ensemble classification technique, distinguishing burns with an accuracy of 93%. An additional human study^64^ performed feature extraction from OCT data and input eight extracted features into a linear classifier based on an ML algorithm to distinguish margins of surgically resected burn tissue from healthy surrounding tissue. For a training set of 34 tissue samples and a test set of 22 tissue samples, the sensitivity and specificity of the classification algorithm were 92% and 90%, respectively.

### 4.2 Ultrasound

Within the past several years, the use of ML burn classification algorithms based on ultrasound data has also been shown. A 2020 report^65^ performed texture analysis of ultrasound images from porcine tissue *ex vivo*. The resulting data was used to train an algorithm for distinguishing between four different burn severities, using a combination of kernel Fisher discriminant analysis and an SVM. This technique provided 93% accuracy for classifying four different burn duration/temperature combinations meant to correspond to superficial-partial thickness, deep partial-thickness, light full-thickness, and deep full-thickness burns. A subsequent porcine study, involving *ex vivo* and postmortem *in situ* skin,^66^ employed a deep CNN with an encoder–decoder network, using ultrasound data (B-mode) as inputs, to distinguish between the four aforementioned burn categories with an accuracy of 99%.

### 4.3 Thermal Imaging

Recent literature has also included the incorporation of thermal imaging data in the infrared wavelength regime into ML algorithms to help classify burn severity. A 2016 study^67^ used data from color images and thermal images in tandem to inform an ML-based classification algorithm that combined multiple techniques, including pattern recognition routines and CNNs. A 2018 report^68^ used thermography with a commercial infrared camera (T400, FLIR System, Wilsonville, OR) to determine the difference in temperature between burns of different treatment groups (amputation, skin graft, and re-epithelialization without grafting) for patients within several days post-burn. An ML algorithm using a random forest technique was developed to predict the burn treatment group (amputation, skin graft, and re-epithelialization) using this temperature data, yielding an accuracy of 85%.

### 4.4 Blood Flow Imaging

Blood flow measurements using coherent light-based techniques (Laser Speckle Imaging, Laser Doppler Imaging) have frequently been employed to identify signatures of burn severity.^75^^–^^81^ Recent research has begun to incorporate data from such measurements into ML algorithms to classify the severity of burns. A recent study^69^ used Laser Speckle Imaging data from a Yorkshire pig burn model as inputs into a CNN to categorize burn depth and predict whether a graft would fail. The algorithm provided accuracies of over 93% for both of these classifications.

### 4.5 Terahertz Imaging

Terahertz (THz) imaging is of interest in a burn severity classification context because, in theory, THz imaging enables wound visualization through gauze bandages. Recent studies have used THz imaging systems as inputs into ML algorithms to diagnose burn severity. Khani et al. and Osman et al.^70^^,^^71^ used a portable time-domain THz scanner to measure three different severities of burns (full thickness, deep partial thickness, and superficial partial thickness) in female Yorkshire^70^ and female Landrace^71^ porcine models. When the THz imaging data were employed to train ML-based classification, the area under the ROC curves for distinguishing between these burn categories ranged from 0.86 to 0.93. In a subsequent study, Khani et al.^72^ used Debye parameters from THz imaging to assess the permittivity of burns in a Landrace pig model, potentially providing a simplified methodology for training ML-based procedures for classifying burn severity.

## 5 Discussion and Conclusions

### 5.1 Summary of Literature to Date, and Current Limitations

**Fig. 7:**
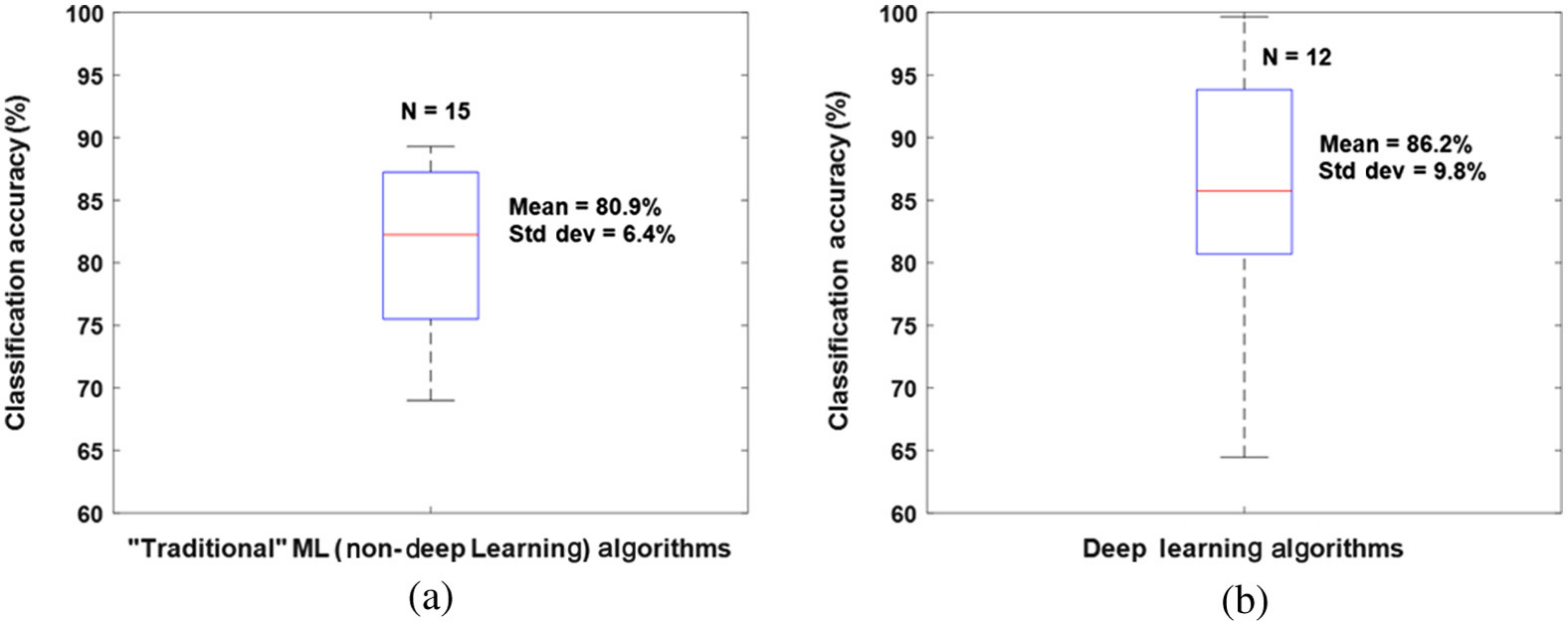
Box plots showing the means, standard deviations, and distributions of reported accuracy values from burn wound classification studies using (a) “traditional” (non-deep learning) ML algorithms and (b) deep learning ML algorithms with digital color images as inputs. Classification results from 15 different “traditional” ML algorithms and 12 different deep learning algorithms were used; the data are from Refs. 19–26, 28, 30, 32, 33, 36, 37, 41–43, and 45–47. Several studies comparing multiple ML algorithms^21^^,^^23^^,^^26^^,^^33^^,^^37^^,^^43^^,^^45^ provided multiple data points that were included in these box plots. Overall, the deep learning algorithms trended toward higher mean accuracy, and the five highest accuracy values were all from deep learning algorithms. However, the deep learning algorithms still had a wide range of reported accuracy values, likely due to the substantial presence of other factors that differed between the studies (e.g., size and composition of dataset; training, validation, and testing procedures; type of ML algorithm employed; types of data pre-processing; and categories used for classification).

Table 1 summarizes the methodologies, classifiers, validation methods, and classification accuracies of the ML methods trained on the imaging modalities described in this review. Over half of these studies used conventional digital color images as inputs to the ML algorithms. A box plot illustrating the distribution of the accuracies of color image-based ML algorithms using “traditional” (non-deep-learning based) ML methods and deep learning approaches is provided in Fig. 7. It is important to note the wide range of reported classification accuracies reported in these studies. The initial purpose of this review was to provide a quantitative comparison between the accuracy of ML algorithms for burn classification using different tissue imaging modalities. However, upon review of the literature, it became clear that the large number of additional variables that are different between the studies make it extremely difficult to objectively identify the most accurate technique(s). These covariates include differences in the preclinical models or patient populations studied; the sizes of the datasets used for training; the specific ML classifiers employed; the training, validation, and testing procedures utilized; and the number and complexity of categories used for classification. Table 1 summarizes several of these covariates, but more work is needed to quantify, in a statistically rigorous manner, the specific effects of each of these different covariates on the reported classification accuracies of the ML algorithms. The rate of growth of this literature and the expansion of different techniques used for obtaining input data to train ML classifiers are depicted in Fig. 1. As the literature in this area continues to expand, it will become even more critical to perform rigorous meta-analyses of the reported results to determine which aspects of the algorithms are most crucial for enabling optimal classification accuracy. For example, the recent trend toward increased use of deep learning approaches appears promising for improving the accuracy of burn wound severity classification, but this hypothesis must be confirmed more rigorously across a wider range of datasets of varying degrees of diversity and complexity.

Furthermore, data from additional emerging technologies such as photoacoustic imaging^82^^,^^83^ may be of significant use for training ML-based burn classification algorithms, motivating additional comparisons with existing literature to assess the effectiveness of these new approaches relative to previously employed imaging modalities. In addition to the potential emergence of new data modalities to provide inputs to ML burn classification algorithms, expanded sets of parameters measured via technologies described in this report may also enhance the input data used for training such algorithms. One example of this possibility is the use of multispectral SFDI (described in Sec. 3.4) to obtain information about the water content of burns with different severities, as an additional input into the ML-based classification procedure. For instance, our group has previously used SFDI to show that water content (denoting edema) can be significantly greater in deep partial-thickness burns than in superficial partial-thickness burns.^84^ This finding is a potentially important inroad into addressing the ongoing clinical need for techniques to more accurately distinguish between these two types of burns, which can appear very similar visually but require very different medical treatment protocols to facilitate healing.

In addition to the need to systematically assess the effects of different components of the ML algorithms on the resulting accuracy, it is also crucial to make sure that the training of the ML classifier is optimal for clinical translation. Multiple studies cited in this report clearly illustrated that certain burn categories were more difficult to accurately classify than others. Potential reasons for this challenge may include biophysical variations between the tissues within a given category or the possibility that certain tissue sites could contain a mixture of different burn categories (e.g., superficial partial thickness and deep partial thickness burn regions) within the same imaged area and sampled tissue volume. Training ML algorithms that are robust in the presence of this level of physiological realism should be prioritized in future studies to facilitate appropriate clinical translation. Establishing clear consensus definitions of each category or one classification system can allow for a better comparison between algorithms and techniques. Also, in clinical settings, it can be a major challenge to obtain enough imaging data from tissue that can unequivocally be classified into each of the “ground-truth” burn severity categories needed to train the ML algorithms. In preclinical studies, the variation in physiology between the different porcine models provides another potential confounding variable that makes direct comparison between studies difficult and may have implications for the effective clinical translation of classifiers.

The “ground-truth” diagnostic information used for training ML-based burn wound classification algorithms is typically provided by clinical observation. It is important to note that, in some cases, the clinical impression itself may not be accurate, especially at time points soon after the creation of the burn. Previous studies have reported that clinical observation can, in some cases, only be accurate for classifying ∼50% to 80% of burn wounds.^13^^–^^16^ Certain critical distinctions (e.g., distinguishing superficial partial-thickness burns, which will heal without skin grafting, from deep partial-thickness burns, which require grafting) can be particularly challenging for clinicians to make promptly and accurately via observation alone.^15^ A recent multi-center initiative^85^ used histology data to train an algorithm for distinguishing between four different burn severities. This algorithm was applied to a dataset of 66 patients (117 burns, 816 biopsies), and following histopathological examination, it was found that 20% of the burns had been mis-classified as severe enough to need grafting. These limitations of current clinical practice provide clear motivation for the development of ML-based classification algorithms but also introduce difficulty in accurately training and validating the algorithms. Furthermore, in many of the reported studies, there was not a clear description of the exact type of “clinical impression” that was used for the “ground-truth” diagnosis/prognosis when training the algorithms. Among the studies that did describe the clinical impression process in more detail, there was notable variation in the time points used for clinical assessment. This absence of a consistent gold standard across studies introduced a further confounding variable that made it difficult to quantitatively compare the accuracies provided by the different imaging modalities.

### 5.2 Conclusions

In this report, we have assembled a comprehensive summary of the literature to date that has used imaging technology to inform ML algorithms to identify burn wounds and classify their severity. Numerous studies indicate that these approaches hold significant promise for helping to inform prompt and accurate clinical decisions as to whether surgical treatment (i.e., grafting) of a burn wound is necessary to enable proper recovery. However, the literature to date is quite disparate, consisting of numerous different combinations of tissue segmentation/tissue classifications, imaging technologies, ML classifiers, and methods for training and validating the algorithms. This wide variance in the literature with respect to multiple different independent variables currently makes it extremely difficult to perform rigorous, systematic, quantitative comparisons between the accuracy of different methodologies with respect to a single independent variable (e.g., imaging modality or ML classifier used). Therefore, to facilitate the optimal translation of these technologies to a wide range of clinical settings, it is crucial for future studies to emphasize the advantages and limitations of their methodologies relative to other reported approaches, with the long-term goal of developing a standardized methodology throughout the field. Incorporation of the most informative of these techniques in a user-friendly and real-time interface is essential for clinical adoption, which would ideally be employed in the operating room.

## Data Availability

As this study is a review of existing literature, the data utilized in this study can be found within the prior publications cited below.
